# Optimization of Deflection of a Big NEO through Impact with a Small One

**DOI:** 10.1155/2014/892395

**Published:** 2014-11-30

**Authors:** Kaijian Zhu, Weiping Huang, Yuncai Wang, Wei Niu, Gongyou Wu

**Affiliations:** ^1^State Key Laboratory of Astronautic Dynamics, Xi'an Satellite Control Center, Xi'an 710043, China; ^2^School of Civil Engineering and Geomatics, Newcastle University, Newcastle upon Tyne NE1 7RU, UK; ^3^School of Management, Xi'an Jiaotong University, Xi'an 710043, China; ^4^Beijing Research Institute of Control and Electronic Technology, Beijing 100038, China

## Abstract

Using a small near-Earth object (NEO) to impact a larger and potentially threatening NEO has been suggested as an effective method to avert a collision with Earth. This paper develops a procedure for analysis of the technique for specific NEOs. First, an optimization method is used to select a proper small body from the database. Some principles of optimality are achieved with the optimization process. Then, the orbit of the small body is changed to guarantee that it flies toward and impacts the big threatening NEO. Kinetic impact by a spacecraft is chosen as the strategy of deflecting the small body. The efficiency of this method is compared with that of a direct kinetic impact to the big NEO by a spacecraft. Finally, a case study is performed for the deflection of the Apophis NEO, and the efficiency of the method is assessed.

## 1. Introduction

Near-Earth object (NEO) impacts have played an important role in the evolution of life, as the Earth has been subjected to frequent impacts since its formation. Humans now recognize not only the historical role of impacts, but also the hazard posed by such events to our modern society. Even though a major impact is unlikely in the short term, one will occur eventually. Numerous methods of NEO deflection have been studied in the literature. The methods can be roughly classified into two kinds: long-duration, low-thrust methods and high-energy impulsive methods. These methods avert collisions either by deflecting NEOs in the means of changing their orbital velocity or by blowing up NEOs in the means of high-energy collisions or explosions.

Long-duration, low-thrust methods include solar ablation [1, 2], laser ablation [3], mass driver [4], gravity tractor [5, 6], solar sail [7, 8], NEO painting [9], enhanced Yarkovsky effect [10], recently the novel long tether and ballast method [11, 12], and high specific impulse rocket [13]. The deflection capability of these methods is relatively limited, and they require a long time to achieve a significant deflection.

High-energy impulsive methods include kinetic impact (with or without explosives) [14], nuclear explosion [15], magnetic flux compression [1], and chemical rocket [9]. If the threatening NEO is sufficiently large, these methods may yield no deflection.

A study about near-Earth objects and deflection studies has been carried out by NASA [16]. It found that if the threatening object is too big or the warning time is not enough for any of the slow-push techniques, a successful deflection using any of the methods listed above is virtually impossible. Deflecting the threatening NEO through impact on a small one may be the only suitable solution.

The main idea of this method is to use a small NEO that is already cataloged in a database to impact the big one. This idea was first introduced in 1992 as a kind of “billiards shot” in a workshop dedicated to the interception of NEOs [17]. A proper small NEO should be chosen from a database and its orbit should be changed in order to achieve the collision. This method can be classified as an impulsive strategy. However, the mass of the small NEO is much higher than that of any artificial objects in space, leading to greater deflection. It must be pointed out that this method requires an accurate dynamical capability, and there must be a body in the database with appropriate orbital parameters and size. Jean et al. studied this method and made a conclusion about characterizing the impact [18]. However, there were no clear ideas suggested about how to choose and deflect the small NEO, and no realistic examples were given.

There are many strategies that can be chosen for orbital transfer of the small NEO: conventional explosion, nuclear explosion, kinetic impact, gravity tractor, and so on. In this study, kinetic impact by a spacecraft is chosen for simplicity. In this paper, physics relationships of collisions in the whole process of this method are analyzed firstly. Then, an optimization method is developed to choose a small NEO as a proper impactor. To analyze the efficiency of this method, its deflection capability is compared with that of a direct impact on the big NEO by a spacecraft. Finally, a case study of deflection of the Apophis NEO is analyzed as a numerical example.

## 2. Physics Relationships in Collisions

There are two collisions during the entire deflection process. The first collision is between the spacecraft and the small NEO, and the second one is the collision between the two NEOs. In a real situation, collisions between a spacecraft and an NEO or between two NEOs are very complicated. The results vary with the bodies' masses, materials, shapes, sizes, and many other factors. The law of conservation of energy is no longer applicable because energy dissipation may happen during the collision. The following assumptions were made in order to permit analysis.

1. The three physical models (a spacecraft and two NEOs) are considered as particles regardless of their shape and size.
2. An accurate dynamic simulation is not used, and the complex chemical changes are ignored.
3. The laws of conservation of mass and conservation of momentum are applicable.

The first collision is analyzed in terms of a central collision between the spacecraft and the small NEO. In the fully inelastic case, the two bodies remain stuck together. Application of the laws of conservation of mass and conservation of momentum yields the final velocity of the small NEO after the collision:

(1) $$\begin{array}{c} V_{s1}=\frac{mv_{\text{need}}+M_{s}V_{s0}}{m+M_{s}}. \end{array}$$

The change in velocity of the small NEO is given by

(2) $$\begin{array}{c} \Delta V_{s}=\frac{m}{M_{s}+m}\left(v_{\text{need}}-V_{s0}\right). \end{array}$$

To analyze the efficiency of this method, similar equations of the collision between a spacecraft and a big NEO can be obtained. The final velocity and the change in velocity of the big NEO after the collision can be written as

(3) Vb1′=mv2+MbVb0′m+Mb,

(4) ΔVb′=mMb+mv2−Vb0′.

Next, the collision between the small NEO and the big NEO is analyzed. Under the assumptions listed above, equations are similar to (1) and (2). The final velocity and the change in velocity of the big NEO after the collision are

(5) Vb1=MsVsf+MbVb0Ms+Mb,

(6) ΔVb=MsMs+MbVsf−Vb0.

Using (1), the velocity of the spacecraft before collision with the small NEO is obtained as

(7) $$\begin{array}{c} v_{\text{need}}=\left(V_{s1}-V_{s0}\right)\frac{M_{s}}{m}+V_{s1}=\Delta V_{s}\cdot \frac{M_{s}}{m}+V_{s1}. \end{array}$$

The change in velocity of the spacecraft before impacting the small NEO, Δ*v* = *v*_need_ − *v*_1_, is given as follows:

(8) $$\begin{array}{c} \Delta v=\Delta V_{s}\cdot \frac{M_{s}}{m}+\left(V_{s1}-v_{1}\right). \end{array}$$

The velocity increment is used for optimization in the following section.

## 3. Optimization of Impactor Selection

### 3.1. Analyses of Velocity Increment (|Δ*v*|)

Parameters associated with the first collision can be optimized to decrease the required energy of the spacecraft. In this study, the transfer process is regarded as a Lambert problem. An optimization method is developed to seek the minimum requirement of the velocity increment. According to the model of a Lambert problem, parameters can be properly chosen to seek minimum energy consumption. The principles of optimality are as follows.

1. Try to ensure that |Δ*v*| = 0; namely, the spacecraft can impact the small NEO directly, without extra energy consumption to change its velocity. If this is possible, it can avoid complex control problems and can improve the success probability of this method; otherwise, try to make it minimum to save energy consumption.
2. Try to make relative velocity of the spacecraft with respect to the Earth when it launched, |Δ*v*_*r*_|, minimum to save energy consumption.

It can be known from (8) that |Δ*v*| is dependent on the value *M*_*s*_/*m*. Setting *y* = Δ*v*,  *x* = *M*_*s*_/*m* > 0,  *k* = Δ*V*_*s*_,  *l* = *V*_*s*1_ − *v*_1_, (8) can be rewritten as

(9) $$\begin{array}{c} y=kx+l. \end{array}$$

Taking the norms of both sides gives

(10) $$\begin{array}{c} \left|y\right|=\sqrt{{\left|k\right|}^{2}x^{2}+2k\cdot lx+{\left|l\right|}^{2}}. \end{array}$$

Setting *a* = |*k*|^2^, *b* = 2*k* · *l*, *c* = |*l*|^2^, *f* = |*y*|^2^, (10) is rearranged to obtain

(11) $$\begin{array}{c} f=ax^{2}+bx+c\;\left(x>0,\;\;a>0,\;\;c>0\right). \end{array}$$

The axis of the symmetry of the quadratic equation is *x*_0_ = −*b*/2*a* and the discriminant is Δ = *b* ^2^ − 4*ac* = 4(*k* · *l*)^2^ − 4*k* ^2^ *l* ^2^ ≤ 0. The mass of the small NEO *M*_*s*_ is much bigger than *m*, so the independent variable of  (11)  *x*, whose value equals *M*_*s*_/*m*, is a large and positive number.

In the first case, if proper parameters, including launch time of a spacecraft from the Earth *t*_0_, transfer time for a spacecraft from the Earth to the small NEO *t*_1_, and transfer time for the small NEO to the big one *t*_2_, are chosen to satisfy *b* < 0 and Δ = 0, the mass of the spacecraft *m* can be chosen to make *M*_*s*_/*m* = −*b*/2*a* and *f* = 0. In this case, |Δ*v*| = 0 means that the spacecraft can impact the small NEO without changing its velocity.

In the second case, if parameters *t*_0_, *t*_1_, and *t*_2_ are chosen to satisfy *b* < 0 and Δ < 0, the mass of the spacecraft can be chosen to make *M*_*s*_/*m* = −*b*/2*a* and *f* = *f*_min⁡_ > 0. This means that the velocity of the spacecraft has to be changed to match *v*_need_, the velocity increment of the spacecraft before collision with the small NEO.

### 3.2. Select Proper NEOs from Database

The mass of the spacecraft is closely associated with the mass of the small NEO. In actual situation, the mass of the spacecraft cannot be large due to the restriction of launch capacity. Therefore, the small NEO cannot be too big if kinetic impact is used to achieve the transfer mission. The restriction will be discussed in the following numeral example.

The difficulty of this method is to seek a proper small NEO as an impactor. Due to the restriction of the launch capability, the mass of the small NEO and velocity increment should be small to make this process possible. Equation (6) can be written into the following form:

(12) $$\begin{array}{c} \Delta V_{b}=\frac{1}{M_{b}/M_{s}+1}\left(V_{sf}-V_{b0}\right). \end{array}$$

The change in velocity of the big NEO is dependent on *M*_*s*_, which is the mass of the small NEO. This means that the deflection of the big NEO increases with the mass of the small NEO. On the other hand, a big candidate requires a large spacecraft impactor, so the candidate cannot be too large for a reasonably sized spacecraft to deflect it. The size of the small NEO is a key variable in this analysis.

The small NEO must have been discovered and must be referenced in a database such as the Near Earth Objects Dynamic Site (NEODyS), which provides the information about all discovered NEOs. In this study, data downloaded from NEODyS is used (see http://newton.dm.unipi.it/neodys/) (2014-05-01). The database contains more than 8400 NEOs and is updated regularly. However, it is not able to sort bodies by size or diameter. Fortunately, the absolute magnitude *H* is related to size. A small *H* means a large size. *H* follows a logarithmic function, *H* = Const + 5Log(size), but here the dependence upon the albedo is not written. Firstly, all the NEOs are sorted by absolute magnitude *H*. Secondly, NEOs which are too large or too small are removed from the database, with only a small part of the NEOs left.

### 3.3. Optimization Method to Find Proper Parameters

A conclusion can be drawn from the above analysis that the choice of the NEO is crucial to this method. An optimization method should be employed to find the best one. The NEOs left in the database as proper candidates are checked one by one.

The optimization process is divided into two steps. The first step is to choose proper candidates from the small part of the NEOs mentioned in Section 3.2, which contains small NEOs with proper size. As the assumption of mass of the spacecraft is 1.0*E*4 kg, there are three optimization variables in the first optimization process: launch time of a spacecraft from the Earth *t*_0_, transfer time for a spacecraft from the Earth to the small NEO *t*_1_, and transfer time for the small NEO to the big one *t*_2_. The objective function is to minimize |Δ*v*_*r*_| + |Δ*v*|. The Matlab built-in function ga is used to find proper initial optimization variables first, and then built-in function fmincon is used to process this optimization problem. The scale range of three variables was given as follows:

(13) $$\begin{array}{c} 0<t_{1}<10\;\text{years},\\ 0<t_{2}<2\;\text{years},\\ 0<t_{3}<2*T_{\text{small}\;\text{NEO}}. \end{array}$$

*T*_small  NEO_ is the orbital period of current checked small NEO of the small part of the NEOs.

Through the first optimization step, candidates with minimum |Δ*v*_*r*_| + |Δ*v*| can be chosen. Check |Δ*v*_*r*_| and |Δ*v*| of these candidates for the qualification of spacecraft capacity. If there is one or more candidates meeting the qualification, do the second step of optimization process. In this step, there are four optimization variables. The first three optimization variables are the same as above, namely, *t*_0_, *t*_1_, and *t*_2_, and the fourth optimization variable is the mass of spacecraft *m*. The primary optimization principle is trying to ensure the spacecraft can impact the small NEO directly, without changes of velocity or make |Δ*v*| as little as possible. Another optimization principle is trying to make relative velocity of the spacecraft to the Earth |Δ*v*_*r*_| minimum to save energy consumption.

Furthermore, the required mass of the spacecraft is dependent on the mass of the small NEO. If relative speed of the spacecraft to the Earth |Δ*v*_*r*_| or speed change of the spacecraft before collision with the small NEO |Δ*v*| is too large, the launch time of a spacecraft from the Earth *t*_0_, transfer time for a spacecraft from the Earth to the small NEO *t*_1_, and transfer time for the small NEO to the big one *t*_2_, or the small NEO chosen as impactor, must be reselected.

If the small NEO is too large or the material, geometry, or other aspects are not appropriate for the first collision, the candidate for this method should also be reselected. Otherwise, a different strategy could be adopted for the orbital transfer of the small NEO, such as a nuclear explosion.

### 3.4. Efficiency Compared with Direct Impact

Based on the analysis above, the efficiency of the double-impact case can be compared to that of the single-impact case. The mass of the spacecraft is much smaller than that of the small NEO. The mass of the small NEO is also much less than that of the big NEO. The ratio Δ*V*_*b*_/Δ*V*_*b*_′ can be derived from (4) and (6):

(14) ξ=ΔVbΔVb′=Mb+mMb+Ms·Msm·Vsf−Vb0v2−Vb0′≈Msm·Vsf−Vb0v2−Vb0′.

The ratio *M*_*s*_/*m*  is very large. In general, the speed of the small NEO relative to the big one |*V*_*sf*_ − *V*_*b*0_| is bigger than that of a spacecraft to the big NEO |*v*_2_ − *V*_*b*0_′|. As a result, the value of  *ξ* is a large number, and the deflection of the big NEO by the impact of a small NEO is much greater than that caused by direct impact by a spacecraft.

## 4. Numerical Examples

This section presents a case study of deflecting asteroid 99942 Apophis, a 300–400 meter-long object that has an impact probability of more than 2% in 2029 [17]. Its mass is about 4.70*e* + 10 kg.

The speed change of Apophis would be about 0.1 m/s due to a collision with a small NEO which is about ten thousand times smaller than it, assuming that the relative speed between them is about 10 km/s. If this velocity increment is imparted several years before the potential collision with Earth, it can prevent the collision.

As mentioned above, because of the restricted mass of the spacecraft, the small NEO chosen as an impactor should be within an appropriate size range. The optimal mass for a spacecraft-impacted NEO is about 1*e* + 06 kg. This makes for a diameter of about 20 m, assuming an average density of  2 g · cm^−3^.

The first step in the search is to exclude NEOs with diameter more than 20 m, which corresponds to an absolute magnitude *H* less than 26.5. Through the initial screening, there are about 400 NEOs left as candidates in the database.

After checking each of the NEOs left in the database using the optimization process, the best NEO as an impactor is 2004HE with *H* = 26.80 and a diameter about 10–20 m.

**Figure 1:**
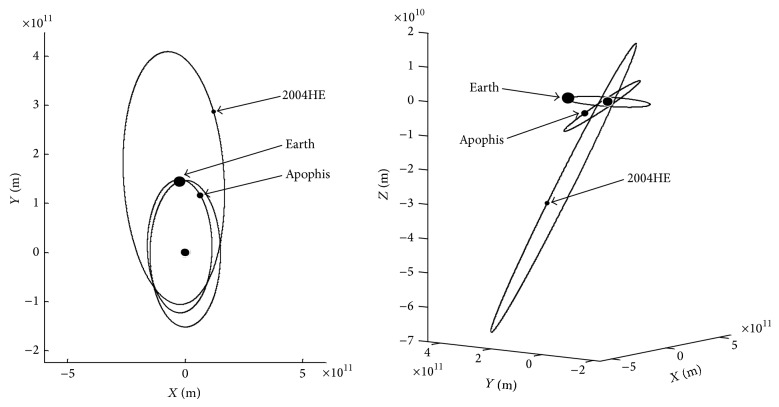
Orbits and positions of Earth, Apophis, and 2004HE.

The orbital parameters of Earth, Apophis, and 2004HE on January 1st, 2012, are shown in Table 1. Their corresponding orbits and positions are shown in Figure 1.

Two groups of parameters, including launch time of a spacecraft from the Earth *t*_0_, transfer time for a spacecraft from the Earth to the small NEO *t*_1_, and transfer time for the small NEO to the big one *t*_2_, are chosen and the result is analyzed.

**Figure 2:**
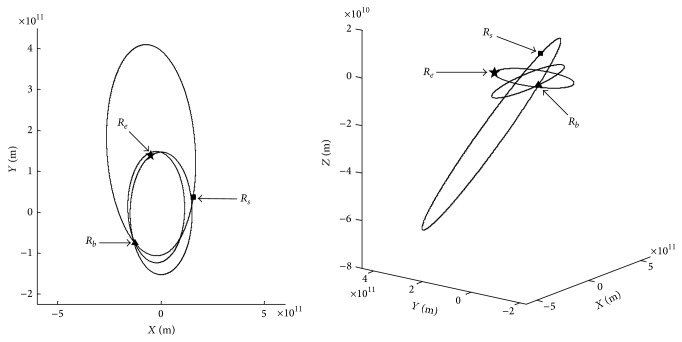
Ends of vectors *R*_*e*_, *R*_*s*_, and *R*_*b*_.

(1) The first group of chosen parameters and their corresponding vectors are shown in Table 2. The ends of vectors are shown in Figure 2. *R*_*e*_ is the vector pointing from the Sun to the Earth at time *t*_0_, *R*_*s*_ is the vector pointing from the Sun to the small NEO at time (*t*_0_ + *t*_1_), and *R*_*b*_ is the vector pointing from the Sun to the big NEO at time (*t*_0_ + *t*_1_ + *t*_2_).

**Figure 3:**
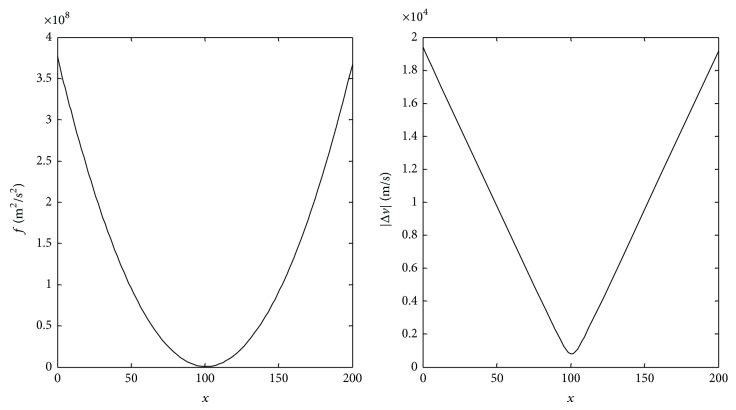
*f* and |Δ*v*|.

Graphs of function *f* = |Δ*v*|^2^ and change in velocity of the spacecraft before impacting the small NEO |Δ*v*| are shown in Figure 3.

The relative speed of the spacecraft to the Earth after it launched is |Δ*v*_*r*_| = 1.55*e* + 03 m/s. If the mass of the spacecraft *m* is chosen to match *M*_*s*_/*m* = −*b*/2*a* = 100.65, being equal to 14,903 kg based on the assumption that the mass of 2004HE is 1.5E6 kg, the change in speed of the spacecraft before the collision is as follows: |Δ*v*|_min⁡_ = 7.99*e* + 02 m/s. This result means that the spacecraft is launched from Earth at time *t*_0_ with a relative speed of 1550 m/s, and it impacts 2004HE at *t*_1_. Its speed should have a change of 799 m/s before collision with the small NEO. Written in a vector form, the velocity variation in the J2000 inertial frame can be described as *k* = [−180.35; −55.97; 38.08] m/s. After a transfer time of *t*_2_, 2004HE impacts Apophis. Due to the collision, the velocity variation of Apophis can be calculated to be Δ*V*_*b*_ = 0.39 m/s, using (2).

**Figure 4:**
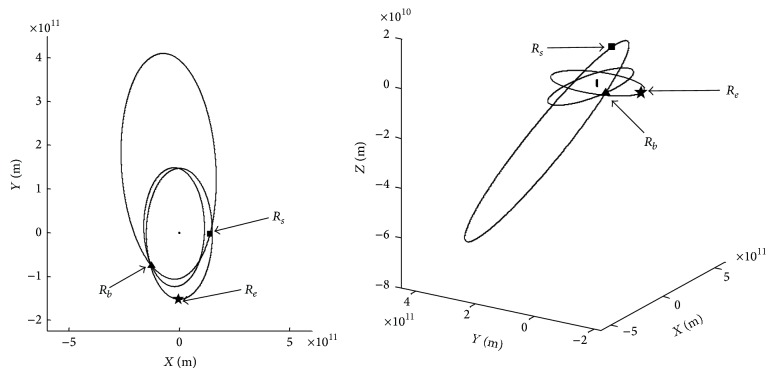
Ends of vectors *R*_*e*_, *R*_*s*_, and *R*_*b*_.

(2) The second group of parameters *t*_0_, *t*_1_, *t*_2_ and their corresponding vectors *R*_*e*_, *R*_*s*_, *R*_*b*_ are shown in Table 3. The ends of vectors are shown in Figure 4.

**Figure 5:**
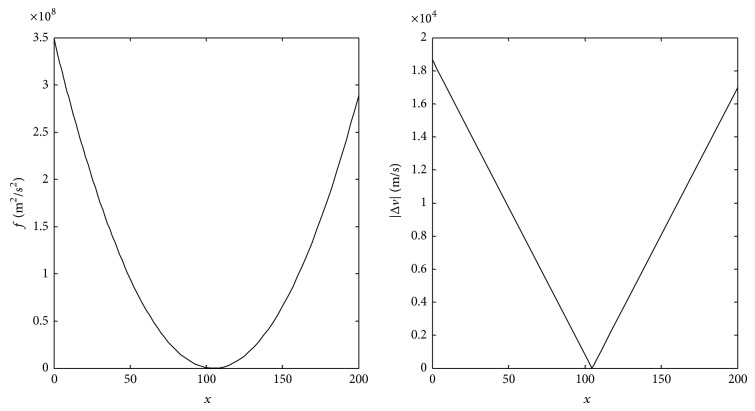
*f* and |Δ*v*|.

Graphs of function*f* = |Δ*v*|^2^ and change in velocity of the spacecraft before impacting the small NEO |Δ*v*| are shown in Figure 5.

The relative speed of the spacecraft to Earth is |Δ*v*_*r*_| = 2.58*e* + 03 m/s. If the mass of the spacecraft *m* is chosen to match *M*_*s*_/*m* = −*b*/2*a* = 104, the change in velocity of the spacecraft before the collision is as follows: |Δ*v*| = 0. The mass can be chosen to be equal to 14, 323 kg based on the assumption that the mass of 2004HE is 1.5E6 kg. This result means that the spacecraft is launched from Earth with a relative speed 2580 m/s, the spacecraft can impact on the small NEO directly without needing to orbit the sun to get into position. Written in vector form, the velocity variation in the J2000 inertial frame can be described as *k* = [−174, −18.03,31.50] m/s. The velocity variation of Apophis due to impact is Δ*V*_*b*_ = 0.39 m/s.

A general conclusion can be drawn: if the velocity of the spacecraft relative to Apophis is equal to that of 2004HE, the variable *x* = *M*_*s*_/*m* is a good evaluation of the efficiency compared with that of a direct impact by a spacecraft. The efficiency of the dual-impact method is about one hundred times of the efficiency of a direct impact by a spacecraft. However, it should be pointed out that a more complete survey of the small NEO candidate is warranted to obtain its physical information and make sure that it will actually suffice for this mission.

## 5. Conclusions

Deflecting a big NEO through impact on a small one is potentially an effective deflection method to avoid a collision with Earth. By developing optimization methods and using fmincon function in Matlab, proper parameters for orbital transfer and proper small NEOs are chosen to realize the collision with threating and big NEOs. The efficiency of this method is much higher compared with that of a direct impact on the big NEO by a spacecraft.

An example of deflection for the Apophis NEO is analyzed using this method. NEO 2004HE is chosen as the impacting body. Two groups of parameters are given, and their results are analyzed, respectively. The results of deflection in this example are satisfactory and show that the efficiency of this method is much higher than that of a direct spacecraft impact on the big NEO. The results suggest that further study of the complex dynamical relationships and other problems associated with this method is warranted.

## Figures and Tables

**Table 1:** Orbital parameters of Earth, Apophis, and 2004HE (Jan. 1, 2012, 00:00).

Body	Earth	Apophis	2004HE
*a* (AU)	1.00*e* + 00	9.22*e* − 01	1.77*e* + 00
*e*	1.67*e* − 02	1.91*e* − 01	6.08*e* − 01
*i* (deg.)	2.11*e* − 03	3.33*e* + 00	9.47*e* + 00
Ω (deg.)	1.57*e* + 02	2.04*e* + 02	2.08*e* + 02
*ω* (deg.)	3.06*e* + 02	1.26*e* + 02	7.93*e* + 01
*M* (deg.)	3.55*e* + 02	6.89*e* + 01	7.39*e* + 01

**Table 2:** *t*_0_, *t*_1_, *t*_2_ and corresponding vectors *R*_*e*_, *R*_*s*_, and *R*_*b*_.

*t*_0_ (s)	*R*_*e*_ (m)	*t*_1_ (s)	*R*_*s*_ (m)	*t*_2_ (s)	*R*_*b*_ (m)
4.11*e* + 08	−4.98*e* + 10 1.38*e* + 11 −3.99*e* + 06	2.56*e* + 07	1.55*e* + 11 3.65*e* + 10 6.89*e* + 09	1.37*e* + 08	−1.26*e* + 11 −7.48*e* + 10 9.16*e* + 08

**Table 3:** *t*_0_, *t*_1_, *t*_2_ and corresponding vectors *R*_*e*_, *R*_*s*_, and *R*_*b*_.

*t*_0_ (s)	*R*_*e*_ (m)	*t*_1_ (s)	*R*_*s*_ (m)	*t*_2_ (s)	*R*_*b*_ (m)
2.04*e* + 08	−4.33*e* + 09 −1.51*e* + 11 5.22*e* + 06	7.80*e* + 06	1.39*e* + 11 −2.51*e* + 09 1.13*e* + 10	1.38*e* + 08	−1.26*e* + 11 −7.50*e* + 10 9.33*e* + 08

## List of Symbols

$M_{b}$: Mass of a big and threatening NEO
$M_{s}$: Mass of a small NEO chosen to impact the big one
m: Mass of a spacecraft chosen to impact an NEO
$\Delta v_{r}$: Relative velocity of the spacecraft to the Earth
$v_{1}$: Velocity of the spacecraft when it meets the small NEO
$v_{2}$: Velocity of the spacecraft when it meets the big NEO (if impacted directly)
$v_{\text{need}}$: Needed velocity of the spacecraft before collision with the small NEO
Δv: $v_{\text{need}}-v_{1}$, velocity change of the spacecraft before its collision
$V_{s0}$: Velocity of the small NEO before collision with the spacecraft
$V_{s1}$: Velocity of the small NEO after collision with the spacecraft
$\Delta V_{s}$: $V_{s1}-V_{s0}$, velocity change of the small NEO under the influence of the collision
$V_{sf}$: Velocity of the small NEO before collision with the big one
$V_{b0}$: Velocity of the big NEO before its collision with the small one
$V_{b1}$: Velocity of the big NEO after its collision with the small one
$\Delta V_{b}$: $V_{b1}-V_{b0}$, velocity change of the big NEO under the influence of the collision
$V_{b0}^{\prime }$: Velocity of the big NEO before collision with a spacecraft (if impacted directly)
$V_{b1}^{\prime }$: Velocity of the big NEO after collision with a spacecraft (if impacted directly)
$\Delta V_{b}^{\prime }$: $V_{b1}^{\prime }-V_{b0}^{\prime }$, velocity change of the big NEO (if impacted directly)
$t_{0}$: Launch time of the spacecraft from the Earth (seconds after Jan. 1st., 2012, 00:00)
$t_{1}$: Transfer time of the spacecraft from the Earth to the small NEO
$t_{2}$: Transfer time of the small NEO to the big one
$R_{e}$: Vector pointing from the Sun to the Earth
$R_{s}$: Vector pointing from the Sun to the small NEO
$R_{b}$: Vector pointing from the Sun to the big NEO.

## References

[1] Adams R. B., Alexander R., Bonometti J. (2004). *Survey of Technologies Relevant to Defense from Near Earth Objects*.

[2] Gong S.-P., Li J.-F., Gao Y.-F. (2011). Dynamics and control of a solar collector system for near Earth object deflection. *Research in Astronomy and Astrophysics*.

[3] Campbell J. W., Phipps C., Smalley L. The impact imperative: laser ablation for deflecting asteroids, meteoroids, and comets from impacting the earth.

[4] Olds J., Charania A., Schaffer M. Mulitiple mass drivers as an option for asteroid deflection missions.

[5] Sckweickart R., Chapman C., Durda D., Hut P. Threat mitigation: the gravity tractor.

[6] Lu E. T., Love S. G. (2005). Gravitational tractor for towing asteroids. *Nature*.

[7] Shengping G., Hexi B., Junfeng L. (2007). Solar sail formation flying around displaced solar orbits. *Journal of Guidance, Control, and Dynamics*.

[8] Gong S., Li J., Bao Yin H. X. (2009). Formation flying solar-sail gravity tractors in displaced orbit for towing near-Earth asteroids. *Celestial Mechanics & Dynamical Astronomy*.

[9] Gritzner C., Häntschel G., Fasoulas S. (2001). Near-Earth object hazard mitigation publication analysis. *Final Report*.

[10] Steven R. C. (2005). Potential impact detection for Near-Earth asteroids: the case of 99942 Apophis (2004 MN4). *Proceedings of the International Astronomical Union*.

[11] French D. B., Mazzoleni A. P. (2009). Asteroid diversion using a long tether and ballast. *Journal of Spacecraft and Rockets*.

[12] Mashayekhi M. J., Misra A. K. (2012). Tether assisted near earth object diversion. *Acta Astronautica*.

[13] Williams B. G., Durda D. D., Scheeres D. J. The B612 mission design.

[14] Adams R. B., Alexander R., Bonemetti J. (2004). *Survey of Technologies Relevant to Defense from Near-Earth Objects*.

[15] Gennery D. Deflection asteroids by means of Standoff nuclear explosions.

[16] NASA (2006). 2006 near-earth object survey and deflection study. *Final Report*.

[17] Canavan G. H., Rather J. D. G. Assessment of current and future technologies.

[18] Jean M. S., Nicolas P., Andrew B. Billiards shot for asteroid deflection.

